# Individual and Combined Effects of Extracellular Polymeric Substances and Whole Cell Components of *Chlamydomonas reinhardtii* on Silver Nanoparticle Synthesis and Stability

**DOI:** 10.3390/molecules24050956

**Published:** 2019-03-08

**Authors:** Ashiqur Rahman, Shishir Kumar, Adarsh Bafana, Si Amar Dahoumane, Clayton Jeffryes

**Affiliations:** 1 Nanobiomaterials and Bioprocessing Laboratory (NABLAB), Dan F. Smith Department of Chemical Engineering, Lamar University, Beaumont, TX 77710, USA; arahman2@lamar.edu (A.R.); skumar1@lamar.edu (S.K.); abafana@lamar.edu (A.B.); 2 School of Biological Sciences and Engineering, Yachay Tech University, Hacienda San José s/n, San Miguel de Urcuquí 100119, Ecuador; sdahoumane@yachaytech.edu.ec; 3 Center for Advances in Water & Air Quality, Lamar University, 211 Redbird Ln, Box 10888, Beaumont, TX 77710-0088, USA

**Keywords:** nanobiomaterials, bionanotechnology, biosynthesis, sustainability, green chemistry

## Abstract

The fresh water microalga *Chlamydomonas reinhardtii* bioreduced Ag^+^ to silver nanoparticles (AgNPs) via three biosynthetic routes in a process that could be a more sustainable alternative to conventionally produced AgNPs. The AgNPs were synthesized in either the presence of whole cell cultures, an exopolysaccharide (EPS)-containing cell culture supernatant, or living cells that had been separated from the EPS-containing supernatant and then washed before being suspended again in fresh media. While AgNPs were produced by all three methods, the washed cultures had no supernatant-derived EPS and produced only unstable AgNPs, thus the supernatant-EPS was shown to be necessary to cap and stabilize the biogenic AgNPs. TEM images showed stable AgNPs were mostly spherical and showed a bimodal size distribution about the size ranges of 3.0 ± 1.3 nm and 19.2 ± 5.0 nm for whole cultures and 3.5 ± 0.6 nm and 17.4 ± 2.6 nm for EPS only. Moreover, selected area electron diffraction pattern of these AgNPs confirmed their polycrystalline nature. FTIR of the as-produced AgNPs identified polysaccharides, polyphenols and proteins were responsible for the observed differences in the AgNP stability, size and shape. Additionally, Raman spectroscopy indicated carboxylate and amine groups were bound to the AgNP surface.

## 1. Introduction

AgNPs have a plethora of applications as reviewed elsewhere [1,2]. However, biosynthetically produced AgNPs, using whole organisms (i.e., plants, algae, bacteria, fungi, etc.) or their derivatives (i.e., proteins, starch, glucose, etc.), could be a more sustainable alternative to conventional methods [3,4,5]. Among the platforms for biosynthetic nanomaterials, algae [6,7] and their extracts [8] are particularly interesting, owing to their simple synthesis conditions and ability to control the shape, size and composition of the produced materials [9]. Thus far, the biosynthesis of AgNPs using microalgae has been explored in four different directions including biomolecules from disrupted cells, cell-free supernatant, harvested whole cells and living cultures [4,8,9,10].

For example, proteins extracted from *Chlorella vulgaris* were used to synthesize silver nanoplates [11] and the pigment C-phycocyanin was found to promote AgNP biosynthesis only under light exposure, suggesting that the process is photon-driven [12]. Additionally, supernatants from numerous microalgae have been used in different forms to synthesize AgNPs [10]. Fine powder from dried biomass of *Spirogyra insignis* [13] and the supernatant from solutions of *Spirulina platensis* powder [14] were used to synthesize AgNPs. Other forms included cell-free filtrate of disrupted cells [15], aqueous cell extract of homogenized cells [16], cell-free supernatant of disrupted cells [17] and cell-free supernatant of harvested cells [12,18] of various species of microalgae.

Besides the extracts, AgNPs can also be produced by using the whole cells of *Plectonema boryanum*, a filamentous cyanobacteria [19]. In this case, the synthesis process took place within the cells, on the surface of the cells and in solution. Cells of *Euglena gracilis* and *Euglena intermedia* were used to intracellularly and extracellularly biosynthesize AgNPs [15]; the former also synthesized AuNPs intracellularly [20,21,22,23]. Several other studies describe the production of AgNPs using harvested whole cells of green microalgae where cells were first separated from culture media then thoroughly washed with and suspended in distilled water [12,24,25,26].

Through a one-step process, AgNPs have been synthesized by adding silver salts directly to the cells maintained under their usual culturing conditions [10]. Cyanobacterial strains *Anabaena flos-aquae, Calothrix pulvinate* and *Leptolyngbya foveolarum* remained viable and produced AgNPs in culture media at AgNO_3_ concentrations up to 0.1 mM [27]. Several other NP syntheses by living cultures were performed with *Chlorophyta* [21,28,29], cyanobacteria [30], *Haptophyta* [28], and *Ochrophyta* [29].

The present article focuses on producing AgNPs using whole living cultures (WLC) of *C. reinhardtii*, extracellular polysaccharide containing supernatant (EPS-S) and living cells that have been washed to remove EPS before being suspended in fresh media (LCFM—living cells fresh media). Although AgNPs have been produced by numerous biological routes, there is a lack of studies that individually determine the effect of intracellular, extracellular and cell-surface effects on AgNP conversion, shape, size and stability. In this study, we used three different AgNO_3_ concentrations to challenge either washed cells, whole living cultures or EPS-containing supernatant to synthesize AgNPs in order to determine how the different cell culture components affect the synthesis process. In addition to studying the surface plasmon resonance (SPR) evolution and the size and shape of the as-produced AgNPs via the three different routes, this article highlights the cell viability and identifies the potential biomolecules responsible for the bioreduction and stability of the AgNPs. The main novelty of this work is that we have designed experiments that discriminate between the effects of the EPS and whole cells in relation to the synthesis and stability. Moreover, we performed the extracellular synthesis using EPS that were harvested without disrupting the cells of *C. reinhardtii*. In fact, a few algal species have shown such extracellular synthesis potential before [26]. This synthesis process can lead to the design of a sustainable and scalable photobioreactor system where *C. reinhardtii* can be recycled to continuously produce EPS that will synthesize stable AgNPs of well-controlled size and shape.

## 2. Results and Discussion

### 2.1. Biosynthesis of AgNPs by C. reinhardtii

**Figure 1:**
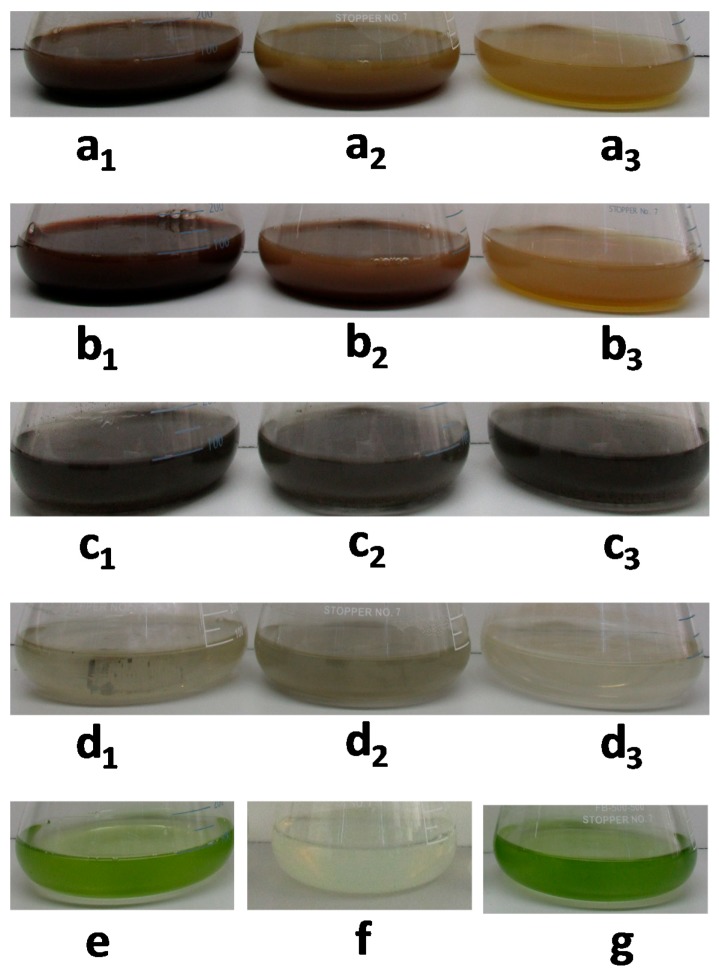
Photographic images after 2 h of cultivation: Erlenmeyer flasks containing AgNO_3_ and (**a**) whole living cultures (WLC); (**b**) extracellular polysaccharide containing supernatant (EPS-S); (**c**) living cells fresh media (LCFM); and (**d**) Bold’s basal medium (BBM) (subscripts 1, 2, 3 denotes three different AgNO_3_ final concentrations, that is, 1.250 mM, 0.625 mM and 0.125 mM, respectively). Also shown are the control Erlenmeyer flasks containing (**e**) WLC; (**f**) EPS-S; and (**g**) LCFM without AgNO_3_.

Images of flasks with WLC, EPS-S and LCFM after 2 h of reaction with aqueous solutions of AgNO_3_ at different initial concentrations are presented in Figure 1. The characteristic color change of the solutions from green to dark brown (Figure 1a–c) indicated the synthesis of AgNPs via the three routes explored [24,31]. The black appearance, in the case of LCFM, is probably due to the intracellular or membrane-bound AgNPs [26]. There was no notable change in the color for pristine culture media, Bold’s basal medium (BBM), after the addition of AgNO_3_ (Figure 1d). The WLC and LCFM cultures (Figure 1e,g, respectively) without AgNO_3_ remained green and no noticeable color change was observed for EPS-S (Figure 1f) in the absence of precursor. However, all the samples shown in Figure 1 were gently hand-shaken before snapshotting to demonstrate the AgNP synthesis and color change; thus, these images are not indicative of the stability results which are discussed in the following sections.

**Figure 2:**
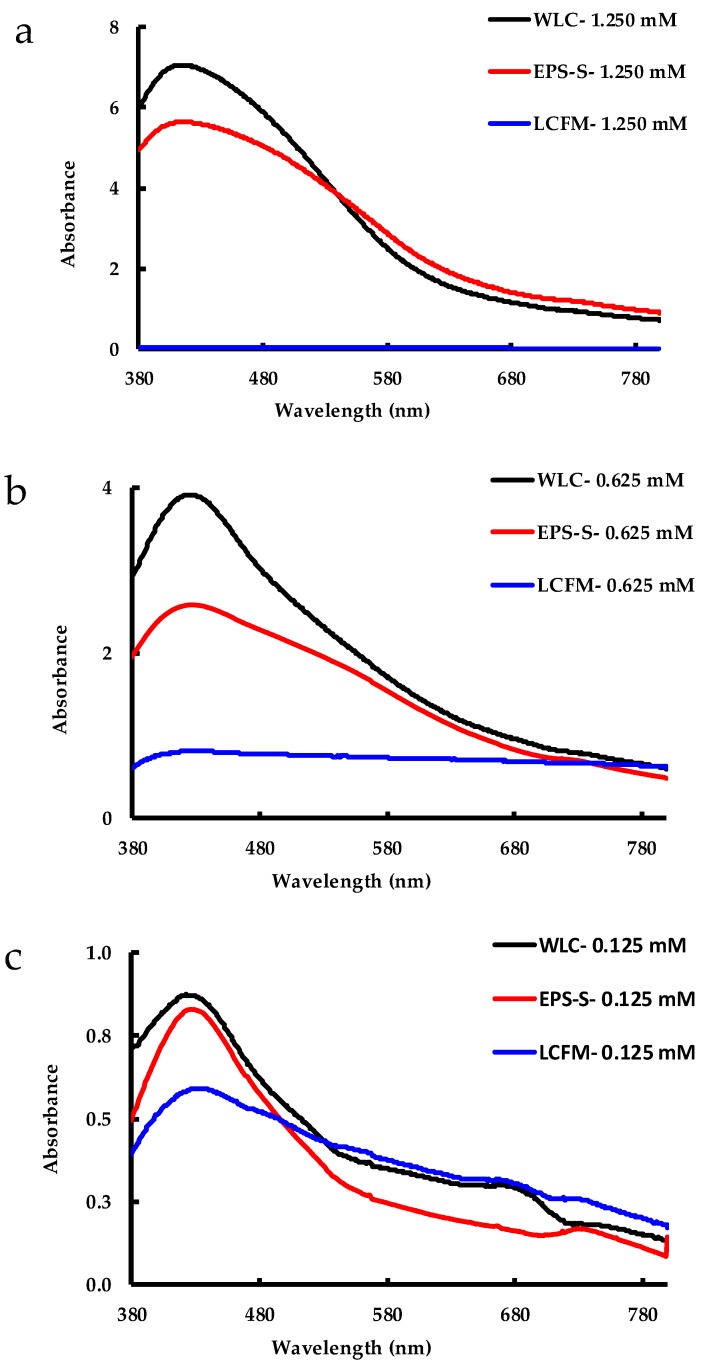
Spectrophotometric measurements for WLC, EPS-S and LCFM after 24 h of reaction at AgNO_3_ concentrations of: (**a**) 1.250 mM; (**b**) 0.625 mM; and (**c**) 0.125 mM.

UV-Visible spectra, recorded 24 h after the addition of AgNO_3_, are presented in Figure 2 for all experiments carried out in the present study. The SPR band, due to the collective oscillation of the electrons at the surface of metallic silver, is observed at ~425 nm in all samples that were challenged by AgNO_3_ [32], except for the LCFM experiment, which is explained later by the reduction of the cell viability. For the three concentrations (Figure 2a–c), the WLC showed a more intense peak than the EPS-S. However, for both WLC and EPS-S, the SPR peaks at higher concentrations (i.e., 1.250 mM and 0.625 mM) tend to be wider than for the lower concentration (i.e., 0.125 mM). In particular, the EPS-S-0.625 shows a shoulder at ~560 nm (Figure 2b). Together, these SPR band features indicate a heterogeneous distribution of AgNP population [31,33] or a strong interaction between the particles [34]. While the LCFM-1.250 mM and LCFM-0.625 mM show a color change (Figure 1c), they showed little to no absorbance at 425 nm (Figure 2a,b) as the spectrophotometric measurements were performed on supernatants, which excluded measurement of unstable AgNPs. However, at 0.125 mM AgNO_3_ (Figure 2c), the AgNP SPR band for LCFM was visible, along with the bands for WLC and EPS-S. This could be due to the formation of lower amounts of AgNPs and their possible stabilization by cell and cell-bound components. Moreover, chlorophyll *a* absorbance bands at ~667 nm in both WLC and LCFM were also observed at this concentration (Figure 2c). We hypothesize that the higher concentration of AgNPs at higher Ag^+^ inputs (i.e., 1.250 mM and 0.625 mM) led to faster aggregation of particles and an earlier onset of sedimentation than the one at lower concentration (i.e., 0.125 mM). To corroborate this, we explained (later in the SPR evolution section) that these AgNPs were indeed stable for a short period before settling down leading their SPR band intensity to decrease.

**Figure 3:**
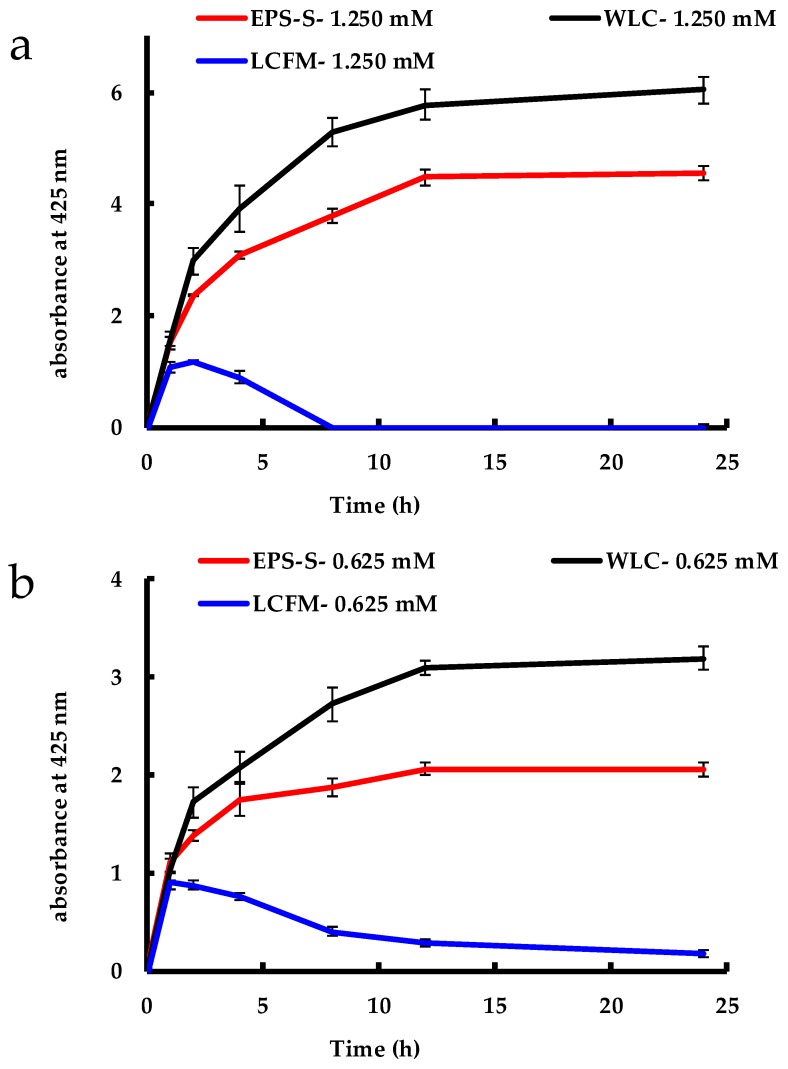

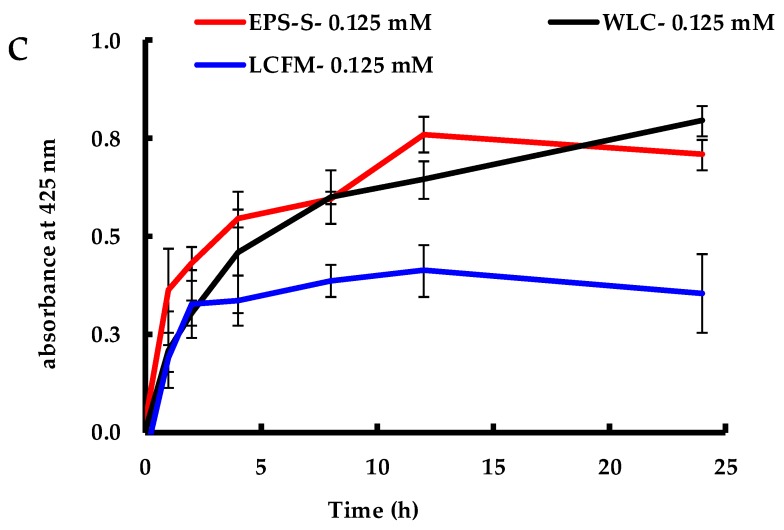
Evolution of AgNP SPR intensity at 425 nm vs. time for WLC, EPS-S and LCFM at AgNO_3_ concentrations of: (**a**) 1.250 mM; (**b**) 0.625 mM; and (**c**) 0.125 mM.

**Figure 4:**
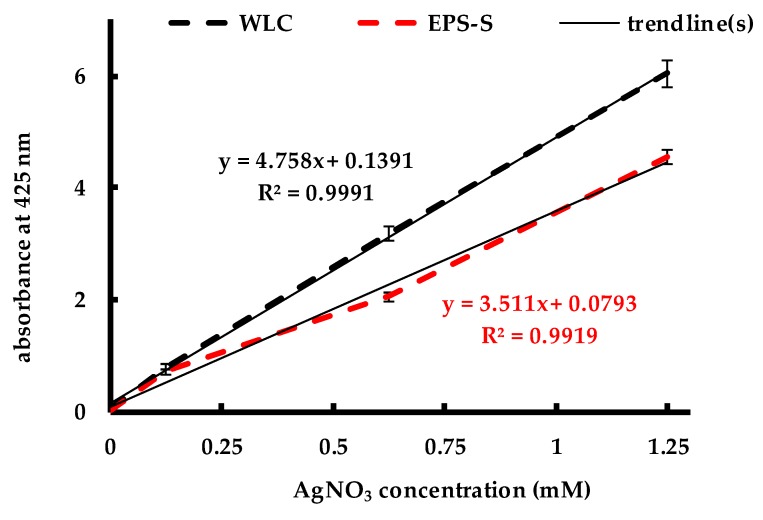
Dependence of maximum stabilized AgNPs as a function of AgNO_3_ input.

The evolution of the SPR band intensity, measured at 425 nm with respect to time, is presented in Figure 3a–c. All the figures show no increase in stabilized AgNPs concentration by WLC and EPS-S beyond 24 h and that AgNPs remained stable over the entire synthesis period. Previous studies using WLC of *C. reinhardtii* show the AgNP synthesis occurs over a period of 92 and 192 h, at 1.250 and 0.625 mM AgNO_3_ concentrations, respectively [31]. However, the contribution of the cell-surface components to the synthesis was not determined. In the current study, higher absorbance values for WLC at all three concentrations of AgNO_3_ indicated that the yield of stable AgNPs was higher for WLC than that of EPS-S. This could be due to additional cell-bound reducing equivalents and NP-stabilizing polysaccharides in the WLC than in the EPS-S, as pointed out in Table 1. Moreover, from the three concentrations explored, it is obvious that the AgNO_3_ was the limiting reactant in the reduction process as the formation of stable AgNPs depended linearly on the initial AgNO_3_ concentration, as shown in Figure 4. This implies that reducing equivalents were excessive and that the AgNP production could be further increased by increasing the amount of added Ag^+^ to the reaction medium.

**Figure 5:**
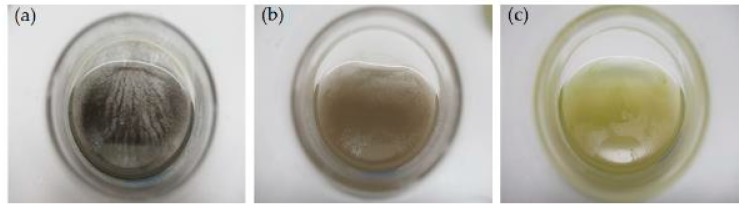
Precipitation of AgNPs at the bottom of the Erlenmeyer flaks (top view) after 24 h of reaction with AgNO_3_: (**a**) LCFM-1.250 mM; (**b**) LCFM-0.625 mM; and (**c**) LCFM-0.125 mM.

In contrast to the synthesis of AgNPs by WLC and EPS-S, the LCFM showed an increase in the SPR band for only the first two hours in LCFM-1.250 and LCFM-0.625 (Figure 3a,b), and the first 12 hours in LCFM-0.125 (Figure 3c). After that, the peak absorbance at 425 nm fell drastically over the next 6 h in LCFM-1.250. The decrease in peak intensity took place in LCFM-0.625 and LCFM-0.125 as well, but gradually over the 24 h experimental period. This reduction in the absorbance can be most likely explained by the fact the produced AgNPs lacked colloidal stability, aggregated and continuously sedimented over the course of the time until they were eliminated from the supernatant [35]. The precipitation of AgNPs is further confirmed by the images shown in Figure 5. In the case of LCFM-1.250 and LCFM-0.625, the precipitates with black macroscopic appearances were clearly visible after 24 h (Figure 5a,b). For LCFM-0.125 (Figure 5c), the precipitate was not visible which could be due to the lower amount of AgNP formation at this concentration as indicated by its UV-Vis spectrum (Figure 3c). Moreover, a green appearance may indicate the presence of chlorophyll *a* from disrupted or whole cells. This fact is corroborated by the presence of chlorophyll *a* absorbance band at ~667 nm in both WLC and LCFM, as discussed earlier (Figure 2c).

### 2.2. Cell Viability

**Figure 6:**
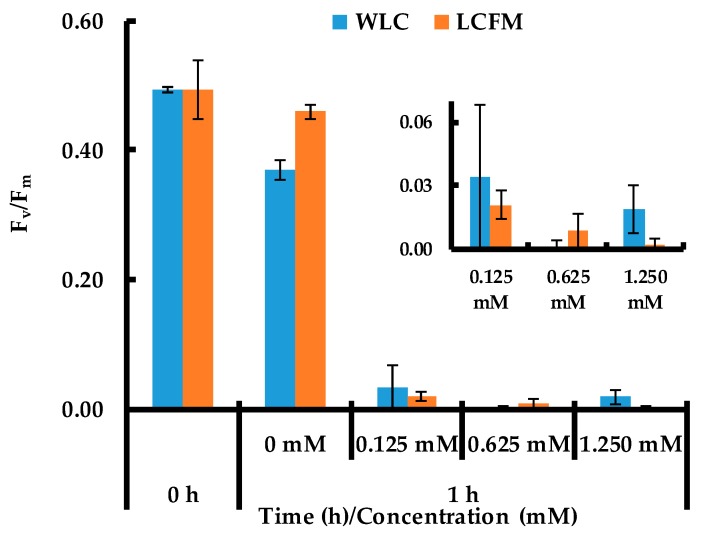
Quantum efficiency of *C. reinhardtii* cells before (0 h) and after (1 h) the addition of AgNO_3_ to WLC and LCFM at different concentrations (the inset magnifies the ~0 F_v_/F_m_ columns by using shorter y-axis scale).

Figure 6 presents the quantum efficiency (F_v_/F_m_) of *C. reinhardtii* cells before and after the addition of AgNO_3_ to WLC and LCFM at different concentrations. Soon after the addition of AgNO_3_ (+1 h), there is at least a 93% and 96% decrease in F_v_/F_m_ values (figures clearly shown in the inset) for WLC and LCFM, respectively, compared to the respective initial values of 0.49 ± 0.01 and 0.49 ± 0.05 (the respective fluorescence signals are presented in Table S1a,b in the Supplementary Materials). In contrast, cells not exposed to AgNO_3_ have F_v_/F_m_ values of 0.37 ± 0.01 for WLC and 0.46 ± 0.01 for LCFM. These results indicate that the cells of *C. reinhardtii* went to a photosynthetically inactive state when exposed to AgNO_3_. This may have stopped the production and release of reducing equivalents and stabilizing agents by the cells. Hence, the AgNPs produced by LCFM were most probably reduced by the cell-bound reducing equivalents but lacked stability because of the absence of free EPS, started aggregating and precipitated over the course of the time. This did not happen in the case of WLC, which had EPS-S that contained the free EPS in addition to the surface bound reducing agents (cf. Table 1).

Moreover, the F_v_/F_m_ values did not show any improvement during the whole experiment. Only WLC-0 and LCFM-0, where no AgNO_3_ was added, maintained intact their photosynthetic activity. These two control experiments indicate a potential damage to the *C. reinhardtii* cells induced by the presence of AgNO_3_. To overcome the toxicity induced by the presence of cationic silver, Dahoumane et al. [21] added fresh media two months after the introduction of AgNO_3_ into the culture and found no viable cells were present. However, our results confirm that this irreversible damage is a fast process taking place immediately after the addition of AgNO_3_. Additionally, almost an equal amount of reduction in quantum efficiencies, at all AgNO_3_ concentrations, indicates that this damage to the *C. reinhardtii* cells is independent of AgNO_3_ concentration, particularly within the range of cell densities to AgNO_3_ concentrations explored in this study.

### 2.3. Morphological Study of the AgNPs

**Figure 7:**
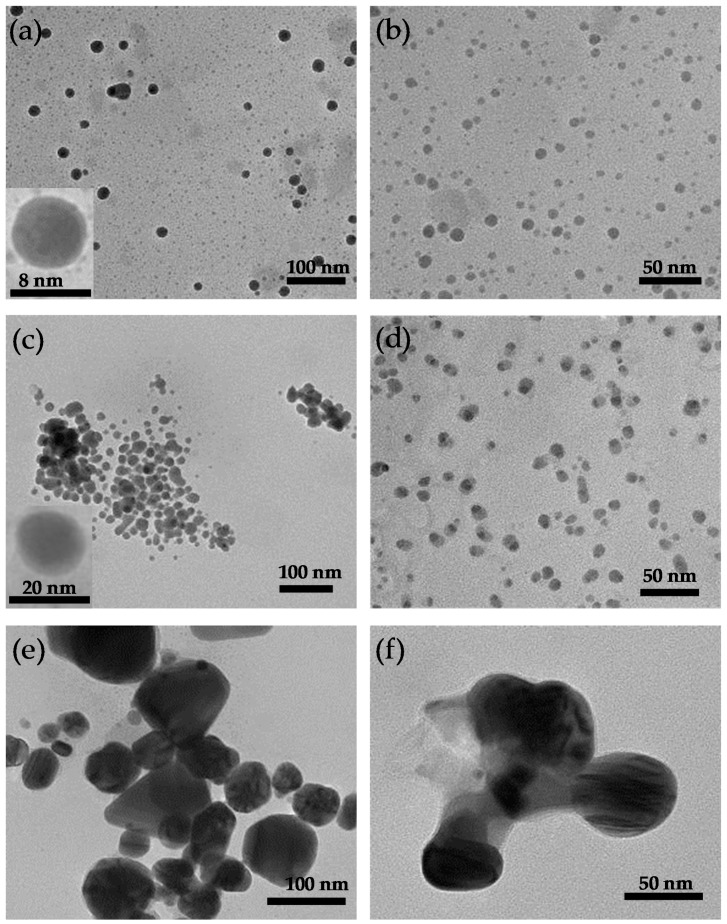
TEM images AgNPs produced by: (**a**) WLC-1.250 mM; (**b**) WLC-0.625 mM; (**c**) EPS-S-1.250 mM; (**d**) EPS-S-0.625 mM; (**e**) LCFM-1.250 mM; and (**f**) LCFM-0.625 mM.

**Figure 8:**
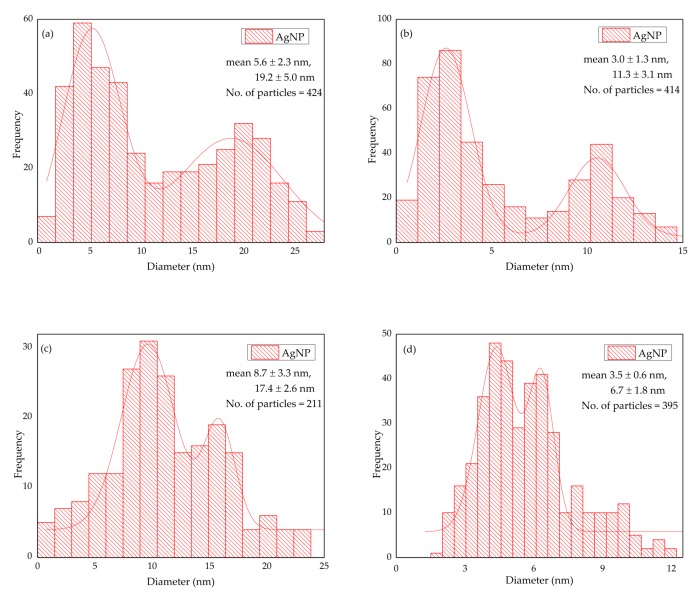
Particle size distribution of AgNPs produced by: (**a**) WLC-1.250 mM; (**b**) WLC-0.625 mM; (**c**) EPS-S-1.250 mM; and (**d**) EPS-S-0.625 mM.

**Figure 9:**
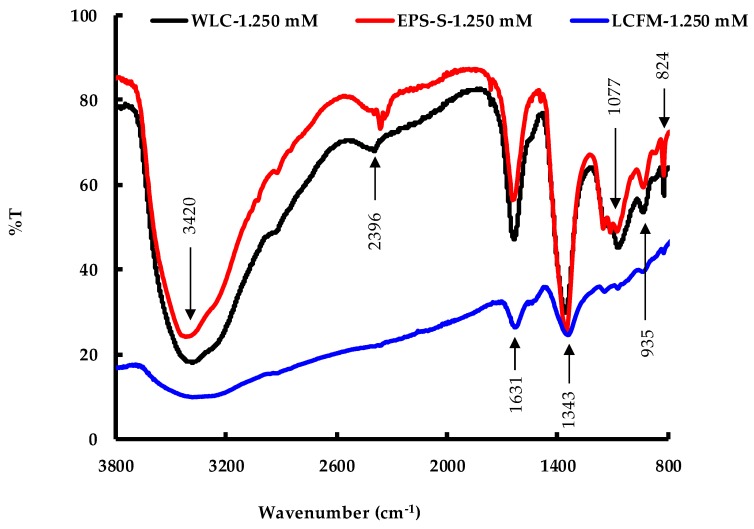
FTIR on the AgNPs produced by WLC-1.250 mM, EPS-S-1.250 mM and LCFM-1.250 mM.

**Figure 10:**
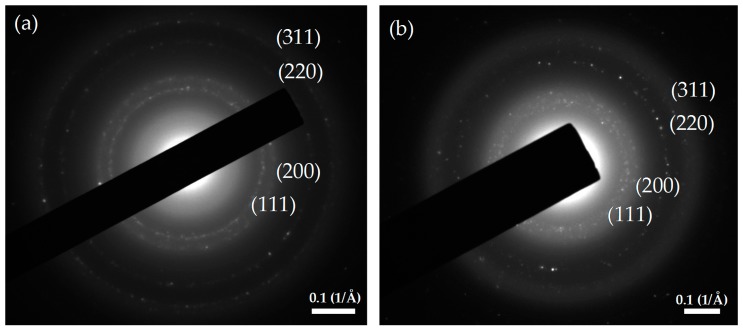
Selected area of electron diffraction (SAED) pattern from one of the AgNPs produced by: (**a**) WLC-1.250 mM; and (**b**) EPS-S-1.250 mM.

Figure 7 shows the TEM micrographs of the AgNPs produced by WLC, EPS-S and LCFM at 1.250 mM and 0.625 mM. A complete visualization of the six micrographs reveals that the AgNPs produced by LCFM (Figure 7e,f), in addition to being randomly shaped, were significantly larger in size compared to the ones produced by WLC (Figure 7a,b) and EPS-S (Figure 7c,d). This could further explain the aggregation and precipitation of these unstable particles that we discussed earlier (see Figure 3). In contrast, WLC and EPS-S produced quasi-similar populations of AgNPs that were well dispersed and mostly spherical in shape. In addition, the insets in Figure 7a,c show AgNPs with lighter edges than their centers, which possibly indicates the attachment of biomolecules on their surfaces [36], hence providing the colloidal stability. Furthermore, the particle size analyses (Figure 8) of WLC and EPS-S samples showed bimodal distributions of AgNPs. These distributions agree with similar results reported recently [31] and further confirm the presence of a heterogenous population of AgNPs as observed in the SPR band (Figure 2). However, the average size of the particles was smaller at 0.625 mM AgNO_3_ concentration compared to the ones at 1.250 mM. At 1.250 mM AgNO_3_ concentration, the average size of AgNPs produced by WLC and EPS-S ranged from 5.6 ± 2.3–19.2 ± 5.0 nm (n = 424) and 8.7 ± 3.3–17.4 ± 2.6 nm (n = 211), respectively, whereas at 0.625 mM AgNO_3_ the range were 3.0 ± 1.3 nm–11.3 ± 3.1 nm (n = 414) and 3.5 ± 0.6–6.7 ± 1.8 nm (n = 395), respectively. Barwal et al. [24] separately used cells and cell free extract of *C. reinhardtii* to produce AgNPs ranging in the size from 5 ± 1 to 15 ± 2 and from 5 ± 1 to 35 ± 5, respectively. However, the wide, bimodal size distribution in the present study (Figure 8) could be from the combined effects of aggregation (Figure 7) and the diversity of biomolecules that synthesized and stabilized the particles (Figure 9, Table 2). Moreover, the AgNPs were polycrystalline as shown in Figure 10 by the SAED pattern [37] recorded from one of the nanoparticles of the respective samples. Altogether, these results show the potential of EPS, released by *C. reinhardtii*, to produce stable AgNPs of similar morphologies to the ones produced by its whole culture. We believe this finding can lead to the design of photobioreactors where the cells can be recycled to continuously produce EPS that will be the central actor in the synthesis of stable AgNPs.

### 2.4. Study of the Biomolecules

FTIR spectroscopy was carried out on the AgNPs in order to determine the potential functional groups of the biomolecules responsible for capping and stabilizing the AgNPs. Figure 9 shows the spectra for AgNP samples produced at 1.250 mM AgNO_3_ via the three routes. Table 2 summarizes the identified biomolecules and their potential sources. For both WLC-1.250 mM and EPS-S-1.250 mM samples, the prominent IR bands were observed at around 3400, 2380, 1640, 1355, 1075, 935 and 825 cm^−1^. These peaks indicated the presence of various biomolecules including polysaccharides, polyphenols, proteins and galacturonic acid. Although almost all these biomolecules were also identified for LCFM-1.250 mM, the intensities were significantly lower. This possibly indicates a lower density of the molecules (shown in Table 2) present on the surface of AgNPs [38] produced by the LCFM route following the removal of the cells from their growth medium, their washing and their re-suspension in a pristine culture medium devoid of any released organic compounds; hence, this explains the aggregation of these particles (cf. see above). Noticeably, Figure 9 reveals the EPS, released by *C. reinhardtii*, to contain sufficient amounts of similar biomolecules as contained in its whole culture. This finding corroborates and explains the potential of EPS to produce stable AgNPs as discussed above.

**Figure 11:**
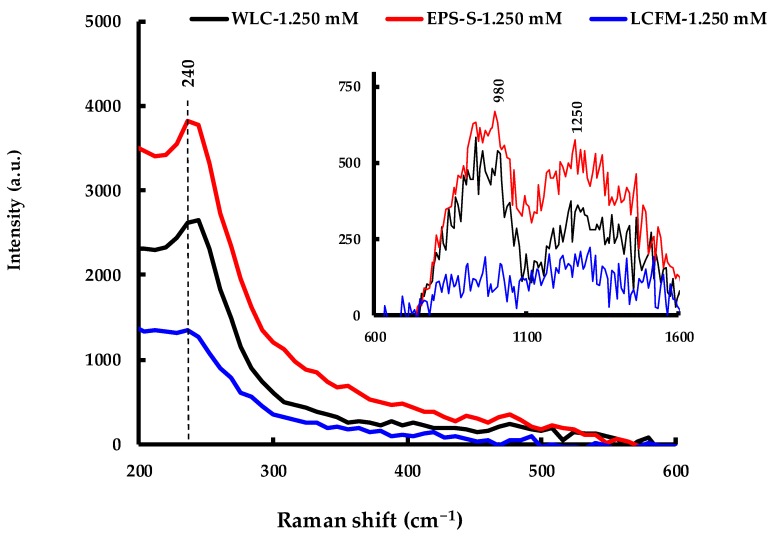
Raman spectroscopy on the AgNPs produced by WLC-1.250 mM, EPS-S-1.250 mM and LCFM-1.250 mM.

In addition to FTIR spectroscopy, Raman spectroscopy was carried out on the same samples in order to identify possible molecular structures interacting with the as-produced AgNPs. The spectrum in Figure 11 shows a strong band at ~240 cm^−1^ that can be assigned to the stretching vibrations of Ag-O and Ag-N bonds [46,47,48]. In terms of intensity, these bands were more prominent in WLC and EPS-S samples than in the LCFM one. While this was the most intense band observed, two other bands, at ~980 cm^−1^ and ~1250 cm^−1^, could also be identified (shown in the inset of Figure 11) that may be attributed to C=CH bending, phenyl ring and C-O-H bending of various biomolecules [46].

## 3. Materials and Methods

### 3.1. Cell Culture Maintenance and Monitoring

#### 3.1.1. Media Preparation

*C. reinhardtii* was cultured in Bold’s basal medium (BBM) which was prepared with several modifications of 3N-BBM+V [49]. The composition of the modified 3N-BBM+V medium was: 430 μmol L^−1^ K_2_HPO_4_, 1.3 mmol L^−1^ KH_2_PO_4_, 300 μmol L^−1^ MgSO_4_·7H_2_O, 2.94 mmol L^−1^ NaNO_3_, 128 μmol L^−1^ CaCl_2_·2H_2_O, 430 μmol L^−1^ NaCl, 132 μmol L^−1^ EDTA, 18 μmol L^−1^ FeSO_4_·7H_2_O, 185 μmol L^−1^ H_3_BO_3_, 4.91 μmol L^−1^ ZnCl_2_, 1.17 μmol L^−1^ MnCl_2_·4H_2_O, 1.01 μmol L^−1^ CuSO_4_·5H_2_O, 280 nmol L^−1^ CoCl_2_·6H_2_O and 794 nmol L^−1^ Na_2_MoO_4_. All chemicals were of analytical grade and purchased from Sigma-Aldrich (St. Louis, MO, USA) or VWR (Radnor, PA, USA); deionized water (DIW) was the solvent. The average pH of the prepared BBM was 6.7 ± 0.2. The BBM was freshly prepared and sterilized by autoclaving at 121 °C and 1 atm gauge for 20 min then allowed to cool for 24 h before being used for sub-culturing.

#### 3.1.2. Sub-Culturing of *C. reinhardtii*

*C. reinhardtii* strains were purchased from the *Chlamydomonas* Resource Center, University of Minnesota, St. Paul, MN, USA. Axenic sub-culturing was done every week in a Labconco Purifier Clean Bench (Labconco Corporation, Kansas City, MO, USA). All materials and BBM were autoclaved prior to use. New generations were prepared by adding 30 mL of previous generation culture to 120 mL of BBM in a 500 mL borosilicate Erlenmeyer flask. The flasks were kept under an average illumination of 69 ± 5 μE m^−2^ s^−1^ provided by cool white LED tubes. The photoperiod was maintained as 16 h/8 h light/dark. The ambient temperature was maintained at 22 ± 1 °C.

### 3.2. Ag^+^ to AgNP Bioreduction Process

Experiments were carried out with whole live cultures (WLC) of *C. reinhardtii*, EPS-containing supernatant separated from the cells the exopolysaccharide-containing cell culture supernatant (EPS-S) and living cells in fresh media (LCFM) that were first washed to remove EPS from the cell culture. Cultures used for all experiments were grown under identical conditions and experiments were performed on 21 days old cultures. The absorbance of extracted chlorophyll *a* (at 663 nm), average cell density and quantum efficiency of these untreated cultures were verified to be consistent in all source cultures prior to all experiments (Figures S1 and S2 in the Supplementary Materials). For WLC, EPS-S and LCFM routes, each experimental flask contained 33% of whole living cultures, 33% of exopolysaccharide-containing cell culture supernatant and 33% re-suspended washed living cells, respectively, and 67% fresh BBM, by volume. Similar dilution was done for EPS-S route to maintain consistency with the previous two experiments.

For the LCFM experiment, 330 mL of whole living culture were centrifuged at 1200× *g* for 5 min. After this, the supernatant was removed and the cell pellet was washed twice with BBM by centrifugation at 1200× *g* for 5 min. After washing, all biomass was collected and transferred into a 1000 mL volumetric flask, which was filled with BBM to make up a final volume of 1000 mL.

Finally, AgNP synthesis was carried out by adding 10 mL AgNO_3_ stock solutions to the cultures to achieve concentrations of 1.250 mmol L^−1^, 0.625 mmol L^−1^ or 0.125 mmol L^−1^ for the three experimental routes. All experiments were carried out in triplicates.

As control experiments, three flasks contained only BBM with the same concentrations of AgNO_3_ as used in the experimental flasks and two duplicate flasks contained 90 mL of cultures with 10 mL of DIW added instead of 10 mL of AgNO_3_ solution.

All flasks were separated by ~5 cm to eliminate any shading of one flask onto another. The average light intensity was 69 ± 5 μE m^−2^ s^−1^. Sampling was done aseptically with 1 mL micropipette in a Labconco Purifier Clean Bench (Labconco Corporation, Kansas City, MO, USA).

### 3.3. Characterization Techniques

#### 3.3.1. Spectrophotometric Characterization

The spectrophotometric characterization was performed using a Cary-100 Bio UV-Vis Spectrophotometer (Agilent Technologies, Santa Clara, CA, USA). Deionized water was the blank for aqueous samples and 89.6% acetone was the blank only when samples contained acetone. All spectrophotometric analyses were carried out in polystyrene cuvettes. The samples were scanned from 380 nm to 800 nm in 1.00 cm path length cuvettes. The crude reaction samples saturated the spectrophotometer, so the samples were diluted 10 times with deionized water, their UV-Vis spectra recorded, multiplied by 10 and reported in the current study. To study the SPR peak evolution, the difference between the maximum peak absorbance at a particular wavelength (λ_max_) and the absorbance at 800 nm (λ_800_) was accounted and plotted vs time.

#### 3.3.2. Pulse Amplitude Modulated (PAM) Fluorometry

The maximum quantum efficiency of photosystem II (F_v_/F_m_), an indication of cell culture health and photonic to chemical energy conversion, was determined by PAM fluorometry using a Z985 Cuvette Aquapen Fluorometer (Qubit Biology Inc., Kingston, ON, Canada), when the Kautsky Induction (OJIP) [50] curves were recorded for 5 s according to the manufacturer’s protocol. The samples were dark adapted for 10 min and diluted 10 times with BBM prior to each measurement [51]. The minimum (F_0_) and maximum (F_m_) fluorescence signals were treated by subtracting the background noise (presented by Table S1c in the Supplementary Materials) obtained from the BBM control experiments.

#### 3.3.3. Morphological and Crystallographic Analyses

Transmission Electron Microscopy (TEM, JEOL Ltd., Tokyo, Japan) was used to characterize AgNP morphology. The reaction media was first filtered using a glass microfiber filter (diameter 25 mm, pore size 1.2 µm). TEM samples were prepared by casting 30 μL of filtrate onto the surface of a PELCO^®^ (Fresno, CA, USA) TEM Grid Carbon Type- B (Ted Pella Inc., 3.05 mm O.D., 400 mesh, 0.4 × 2 mm single slot Cu) and air dried for 24 h. The TEM analysis used a JOEL JEM-1400 Plus Transmission Electron Microscope (120 kV, 1 kV step, 69 μA beam current, 100 kX magnification, spot size 1) equipped with embedded Scanning Transmission Electron Microscopy (STEM, JEOL Ltd., Tokyo, Japan) and selected area electron diffraction (SAED).

#### 3.3.4. Fourier-Transform Infrared Spectroscopy (FTIR)

FTIR analysis of the dried AgNPs was carried out through the potassium bromide (KBr) pellet method [52,53]. The reaction mixture was first centrifuged at 6000× *g* for 10 min and the supernatant was filtered using a glass microfiber filter (diameter 25 mm, pore size 1.2 µm). The filtration step was repeated twice to ensure the removal of uncoordinated biological materials. Then, the AgNP suspension was freeze-dried using a Labconco Freezone Freeze Dry System (Labconco Corporation, Kansas City, MO, USA). Finally, 4 mg dried AgNPs were mixed with KBr in a 1:50 ratio to prepare the pellet. The FTIR spectrum on the pellet was recorded using a Thermo Scientific Nicolet iS10 FTIR Spectrometer (Thermo-Scientific, Waltham, MA, USA). The machine operated in transmittance mode at a resolution of 4 cm^−1^, the pellets were scanned in the spectral ranges of 4000–400 cm^−1^ and the results were presented in percent transmittance (%T).

#### 3.3.5. Raman Spectroscopy

Raman spectroscopy on the AgNP solutions were performed using Perkin Elmer Raman Flex 400F (PerkinElmer, Waltham, MA, USA). The samples were taken in quartz cells and were irradiated by a 350 mW, 785 nm laser delivering 100 mW at the sample. Raman spectra were recorded within a spectral range of 100–2004 cm^−1^ with a data interval of 4 cm^−1^.

### 3.4. Statistical Techniques

All the experiments were carried out in triplicates and averaged data were presented with error bars equal to one standard deviation. The standard deviations were calculated using the Microsoft Excel software program (version 16.0, Redmond, WA, USA). For particle size distribution analyses, frequency histograms were plotted from the raw particle size data obtained by ImageJ software (version 1.8.0), developed by the National Institutes of Health (NIH), Bethesda, MD, USA. The following equation was used to calculate the bin width for the histograms, where N is the square root of the number of data values:

(1) $$\mathrm{Bin}\;\mathrm{width}\;=\;\frac{\mathrm{maximum}\;\mathrm{value}\;-\;\mathrm{minimum}\;\mathrm{value}}{\mathrm{number}\;\mathrm{of}\;\mathrm{bins}\;(N)}$$

Further, the histograms were curve-fitted using Gaussian peak function in OriginPro software (version 9.0, Northampton, MA, USA). The multiple peak fit function was used in the case of fitting bimodal distribution.

## 4. Conclusions and Future Perspectives

The current study shows the potential of extracellular AgNP production by the biomolecules released by *C. reinhardtii* as a part of their metabolism. A linear relationship between the formation of stabilized AgNPs and the AgNO_3_ input was observed, but only when EPS was present during the synthesis. Also, because the LCFM culture had no EPS, but still produced AgNPs, this indicates that surface bound reducing equivalents were able to convert Ag^+^ to Ag^0^, but EPS was required for stability as observed in the EPS and WLC synthesis. These AgNPs, produced by WLC and EPS, were spherical in shape and showed bimodal size distribution. Furthermore, the potential biomolecules, namely polysaccharides, polyphenols, proteins, etc., and their interaction with the AgNPs were identified. However, it is crucial to quantify the amount and potential of these biomolecules in the biosynthesis processes to completely understand the underlying mechanism and to scale-up the process to a photobioreactor system. Nevertheless, the current study shows the potential of producing small and stable AgNPs by both living cultures of *C. reinhardtii* and its cell-released EPS that can lead to the design of photobioreactors where the cells can be recycled to continuously produce EPS that will synthesize stable AgNPs.

## Figures and Tables

**Table 1:** The presence of available biological sources in the different biosynthesis routes.

Route	Available Biological Sources of Reducing and Stabilizing Agents		
	Whole Cells	Free EPS	Cell-Bound Reducing Equivalents
WLC	✓	✓	✓
EPS-S	✕	✓	✕
LCFM	✓	✕	✓
BBM	✕	✕	✕

**Table 2:** Functional groups identified by FTIR and their potential sources.

Band (cm^−1^)	Functional Group	Possible Source	Reference
3400	the O-H stretching vibration	polysaccharides or polyphenols	[39,40,41,42]
2380	C-H vibration	some biomolecules	[43]
1640	stretching vibration of the amide ([NH]C=O) group	protein	[39,41,42]
1355	stretching vibration COO^−^ ion	galacturonic acid	[44,45]
1075	C-O stretching vibration	phenolic group	[39]
935	stretching vibration of C-O-C	polysaccharides	[39]
825	C-H stretching vibration	alkene group	[39]

## Supplementary Materials

The following are available online. Figure S1. Cell density of *C. reinhardtii* cultures used in the three synthesis routes; Figure S2. *Chl*-a absorbance at 663 nm and quantum efficiency of *C. reinhardtii* cultures used in the three synthesis routes; Table S1a. Fluorescence signals (10× diluted) and quantum efficiencies (Q.E.) for WLC before and 1 h after the addition of AgNO_3_; Table S1b. Fluorescence signals (10× diluted) and quantum efficiencies (Q.E.) for LCFM before and 1 h after the addition of AgNO_3_; Table S1c. Background noise from BBM control experiments 1 h after the addition of AgNO_3_.

## References

[1] De Matteis V., Cascione M., Toma C., Leporatti S. (2018). Silver nanoparticles: Synthetic routes, in vitro toxicity and theranostic applications for cancer disease. Nanomaterials.

[2] Marin S., Mihail Vlasceanu G., Elena Tiplea R., Raluca Bucur I., Lemnaru M., Minodora Marin M., Mihai Grumezescu A. (2015). Applications and toxicity of silver nanoparticles: A recent review. Curr. Top. Med. Chem..

[3] Bafana A., Kumar S.V., Temizel-Sekeryan S., Dahoumane S.A., Haselbach L., Jeffryes C.S. (2018). Evaluating microwave-synthesized silver nanoparticles from silver nitrate with life cycle assessment techniques. Sci. Total Environ..

[4] Dahoumane S.A., Mechouet M., Wijesekera K., Filipe C.D.M., Sicard C., Bazylinski D.A., Jeffryes C. (2017). Algae-mediated biosynthesis of inorganic nanomaterials as a promising route in nanobiotechnology—A review. Green Chem..

[5] Kumar S.V., Bafana A.P., Pawar P., Rahman A., Dahoumane S.A., Jeffryes C.S. (2018). High conversion synthesis of <10 nm starch-stabilized silver nanoparticles using microwave technology. Sci. Rep..

[6] Dahoumane S.A., Djediat C., Yéprémian C., Couté A., Fiévet F., Coradin T., Brayner R. (2012). Recycling and adaptation of *Klebsormidium flaccidum* microalgae for the sustained production of gold nanoparticles. Biotechnol. Bioeng..

[7] Jeffryes C., Agathos S.N., Rorrer G. (2015). Biogenic nanomaterials from photosynthetic microorganisms. Curr. Opin. Biotechnol..

[8] Dahoumane S.A., Wujcik E.K., Jeffryes C. (2016). Noble metal, oxide and chalcogenide-based nanomaterials from scalable phototrophic culture systems. Enzyme Microb. Technol..

[9] Dahoumane S.A., Jeffryes C., Mechouet M., Agathos S.N. (2017). Biosynthesis of inorganic nanoparticles: A fresh look at the control of shape, size and composition. Bioengineering.

[10] Dahoumane S.A., Mechouet M., Alvarez F.J., Agathos S.N., Jeffryes C. (2016). Microalgae: An outstanding tool in nanotechnology. Bionatura.

[11] Xie J., Lee J.Y., Wang D.I.C., Ting Y.P. (2007). Silver nanoplates: From biological to biomimetic synthesis. ACS Nano.

[12] Patel V., Berthold D., Puranik P., Gantar M. (2015). Screening of cyanobacteria and microalgae for their ability to synthesize silver nanoparticles with antibacterial activity. Biotechnol. Rep..

[13] Castro L., González F., Blázquez M.L., Muñoz J.A., Ballester A. (2013). Biological synthesis of metallic nanoparticles using algae. IET Nanobiotechnol..

[14] Suganya K.S.U., Govindaraju K., Kumar V.G., Dhas T.S., Karthick V., Singaravelu G., Elanchezhiyan M. (2015). Size controlled biogenic silver nanoparticles as antibacterial agent against isolates from HIV infected patients. Spectrochim. Acta A.

[15] Li Y., Tang X., Song W., Yan X., Ren Q., Liu X., Jin C., Zhu L. (2015). Biosynthesis of silver nanoparticles using *Euglena gracilis*, *Euglena intermedia* and their extract. IET Nanobiotechnol..

[16] Jena J., Pradhan N., Dash B.P., Panda P.K., Mishra B.K. (2015). Pigment mediated biogenic synthesis of silver nanoparticles using diatom *Amphora* sp. and its antimicrobial activity. J. Saudi Chem. Soc..

[17] Durán N., Marcato P.D., Conti R.D., Alves O.L., Costa F., Brocchi M. (2010). Potential use of silver nanoparticles on pathogenic bacteria, their toxicity and possible mechanisms of action. J. Braz. Chem. Sci..

[18] Sudha S.S., Rajamanickam K., Rengaramanujam J. (2013). Microalgae mediated synthesis of silver nanoparticles and their antibacterial activity against pathogenic bacteria. Indian J. Exp. Biol..

[19] Lengke M.F., Fleet M.E., Southam G. (2007). Biosynthesis of silver nanoparticles by filamentous cyanobacteria from a silver(I) nitrate complex. Langmuir.

[20] Dahoumane S.A., Yéprémian C., Djédiat C., Couté A., Fiévet F., Coradin T., Brayner R. (2016). Improvement of kinetics, yield, and colloidal stability of biogenic gold nanoparticles using living cells of *Euglena gracilis* microalga. J. Nanopart. Res..

[21] Dahoumane S.A., Wijesekera K., Filipe C.D.M., Brennan J.D. (2014). Stoichiometrically controlled production of bimetallic gold-silver alloy colloids using micro-alga cultures. J. Colloid Interfaces Sci..

[22] Dahoumane S.A., Yéprémian C., Djédiat C., Couté A., Fiévet F., Coradin T., Brayner R. (2014). A global approach of the mechanism involved in the biosynthesis of gold colloids using micro-algae. J. Nanopart. Res..

[23] Dahoumane S.A., Djediat C., Yéprémian C., Couté A., Fiévet F., Coradin T., Brayner R. (2012). Species selection for the design of gold nanobioreactor by photosynthetic organisms. J. Nanopart. Res..

[24] Barwal I., Ranjan P., Kateriya S., Yadav S.C. (2011). Cellular oxido-reductive proteins of *Chlamydomonas reinhardtii* control the biosynthesis of silver nanoparticles. J. Nanobiotechnol..

[25] Jena J., Pradhan N., Nayak R.R., Dash B.P., Sukla L.B., Panda P.K., Mishra B.K. (2014). Microalga *Scenedesmus* sp.: A potential low-cost green machine for silver nanoparticle synthesis. J. Microbiol. Biotechnol..

[26] Satapathy S., Shukla S.P., Sandeep K.P., Singh A.R., Sharma N. (2015). Evaluation of the performance of an algal bioreactor for silver nanoparticle production. J. Appl. Phycol..

[27] Brayner R., Barberousse H., Hemadi M., Djedjat C., Yéprémian C., Coradin T., Livage J., Fiévet F., Couté A. (2007). Cyanobacteria as bioreactors for the synthesis of Au, Ag, Pd, and Pt nanoparticles via an enzyme-mediated route. J. Nanosci. Nanotechnol..

[28] Merin D.D., Prakash S., Bhimba B.V. (2010). Antibacterial screening of silver nanoparticles synthesized by marine micro algae. Asian Pac. J. Trop. Med..

[29] Mohseniazar M., Barin M., Zarredar H., Alizadeh S., Shanehbandi D. (2011). Potential of microalgae and lactobacilli in biosynthesis of silver nanoparticles. BioImpacts.

[30] Roychoudhury P., Gopal P.K., Paul S., Pal R. (2016). Cyanobacteria assisted biosynthesis of silver nanoparticles—A potential antileukemic agent. J. Appl. Phycol..

[31] Rahman A., Kumar S., Bafana A., Dahoumane S.A., Jeffryes C. (2019). Biosynthetic conversion of Ag^+^ to highly stable Ag^0^ nanoparticles by wild type and cell wall deficient strains of *Chlamydomonas reinhardtii*. Molecules.

[32] Cheviron P., Gouanvé F., Espuche E. (2014). Green synthesis of colloid silver nanoparticles and resulting biodegradable starch/silver nanocomposites. Carbohyd. Polym..

[33] Aswathy B., Avadhani G.S., Sumithra I.S., Suji S., Sony G. (2011). Microwave assisted synthesis and UV–Vis spectroscopic studies of silver nanoparticles synthesized using vanillin as a reducing agent. J. Mol. Liq..

[34] Sharma S., Thakur M., Deb M.K. (2007). Synthesis of silver nanoparticles using N 1, N 2-diphenylbenzamidine by microwave irradiation method. J. Exp. Nanosci..

[35] Jacob J.A., Kapoor S., Biswas N., Mukherjee T. (2007). Size tunable synthesis of silver nanoparticles in water–ethylene glycol mixtures. Colloid Surface A.

[36] Li S., Shen Y., Xie A., Yu X., Qiu L., Zhang L., Zhang Q. (2007). Green synthesis of silver nanoparticles using *Capsicum annuum* L. extract. Green Chem..

[37] Ahmad A., Mukherjee P., Senapati S., Mandal D., Khan M.I., Kumar R., Sastry M. (2003). Extracellular biosynthesis of silver nanoparticles using the fungus *Fusarium oxysporum*. Colloid Surface B.

[38] Bhardwaj A.K., Shukla A., Maurya S., Singh S.C., Uttam K.N., Sundaram S., Singh M.P., Gopal R. (2018). Direct sunlight enabled photo-biochemical synthesis of silver nanoparticles and their Bactericidal Efficacy: Photon energy as key for size and distribution control. J. Photochem. Photobiol. B.

[39] Arévalo-Gallegos A., Garcia-Perez J.S., Carrillo-Nieves D., Ramirez-Mendoza R.A., Iqbal H.M., Parra-Saldívar R. (2018). *Botryococcus braunii* as a bioreactor for the production of nanoparticles with antimicrobial potentialities. Int. J. Nanomed..

[40] Das J., Velusamy P. (2013). Antibacterial effects of biosynthesized silver nanoparticles using aqueous leaf extract of *Rosmarinus officinalis* L.. Mater. Res. Bull..

[41] Rao M.D., Pennathur G. (2017). Green synthesis and characterization of cadmium sulphide nanoparticles from *Chlamydomonas reinhardtii* and their application as photocatalysts. Mater. Res. Bull..

[42] Vamanu E., Ene M., Biță B., Ionescu C., Crăciun L., Sârbu I. (2018). In vitro human microbiota response to exposure to silver nanoparticles biosynthesized with mushroom extract. Nutrients.

[43] Stuart B.H. (2006). Infrared spectroscopy of biological applications: An overview. Encyclopedia of Analytical Chemistry: Applications, Theory and Instrumentation.

[44] Bafana A. (2013). Characterization and optimization of production of exopolysaccharide from *Chlamydomonas reinhardtii*. Carbohyd. Polym..

[45] Giordano M., Kansiz M., Heraud P., Beardall J., Wood B., McNaughton D. (2001). Fourier transform infrared spectroscopy as a novel tool to investigate changes in intracellular macromolecular pools in the marine microalga *Chaetoceros muellerii* (Bacillariophyceae). J. Phycol..

[46] Biswas N., Kapoor S., Mahal H.S., Mukherjee T. (2007). Adsorption of CGA on colloidal silver particles: DFT and SERS study. Chem. Phys. Lett..

[47] Chowdhury J., Ghosh M. (2004). Concentration-dependent surface-enhanced Raman scattering of 2-benzoylpyridine adsorbed on colloidal silver particles. J. Colloid Interfaces Sci..

[48] Kora A.J., Sashidhar R.B., Arunachalam J. (2012). Aqueous extract of gum olibanum (*Boswellia serrata*): A reductant and stabilizer for the biosynthesis of antibacterial silver nanoparticles. Process Biochem..

[49] Grama B.S., Chader S., Khelifi D., Agathos S.N., Jeffryes C. (2014). Induction of canthaxanthin production in a Dactylococcus microalga isolated from the Algerian Sahara. Bioresour. Technol..

[50] Ritchie R.J. (2008). Fitting light saturation curves measured using modulated fluorometry. Photosynth. Res..

[51] Grama B.S., Agathos S.N., Jeffryes C.S. (2016). Balancing photosynthesis and respiration increases microalgal biomass productivity during photoheterotrophy on glycerol. ACS Sustain. Chem. Eng..

[52] Banerjee P., Satapathy M., Mukhopahayay A., Das P. (2014). Leaf extract mediated green synthesis of silver nanoparticles from widely available Indian plants: Synthesis, characterization, antimicrobial property and toxicity analysis. Bioresour. Bioprocess.

[53] Jena J., Pradhan N., Dash B.P., Sukla L.B., Panda P.K. (2013). Biosynthesis and characterization of silver nanoparticles using microalga *Chlorococcum humicola* and its antibacterial activity. Int. J. Nanomater. Biostruct..

