# Supplementary material for: Individual and Combined Effects of Extracellular Polymeric Substances and Whole Cell Components of Chlamydomonas reinhardtii on Silver Nanoparticle Synthesis and Stability
Source: Molecules. 2019 Mar 8;24(5):956. doi: 10.3390/molecules24050956 (PMC6429613; doi:10.3390/molecules24050956)
Supplement: Supplementary file 1 [file molecules-24-00956-s001.pdf]

Supplementary Materials

Individual and Combined Effects of Extracellular Polymeric Substances and Whole Cell Components of *Chlamydomonas reinhardtii* on Silver Nanoparticle Synthesis and Stability

Ashiqur Rahman [1], Shishir Kumar [1], Adarsh Bafana [1], Si Amar Dahoumane [2], Clayton Jeffryes [1,3,*]

[1] Nanobiomaterials and Bioprocessing Laboratory (NABLAB), Dan F. Smith Department of Chemical Engineering, Lamar University, Beaumont, TX 77710, USA; arahman2@lamar.edu (A.R.), skumar1@lamar.edu (S.K.), abafana@lamar.edu (A.B.), cjeffryes@lamar.edu (C.J.)

[2] School of Biological Sciences and Engineering, Yachay Tech University, Hacienda San José s/n, San Miguel de Urcuquí 100119, Ecuador.; sdahoumane@yachaytech.edu.ec (SA.D)

[3] Center for Advances in Water & Air Quality, Lamar University, 211 Redbird Ln, Box 10888, Beaumont, TX 77710-0088, USA

* Correspondence: cjeffryes@lamar.edu; Tel.: +1-409-880-7654

Contents:

1. Cell density (Figure S1)

2. Cell culture chlorophyll *a* composition (Figure S2)

3. Fluorescence signals and quantum efficiencies (Table S1a, Table S1b, Table S1c)

1. Cell density

10 μL-sample was cast on a hemocytometer (0.0025 mm², 0.1000 mm) and the cell culture density was determined by counting the number of cells per volume as observed in a Nikon Labophot-2 Light-microscope (Niko Inc., Minato, Tokyo, Japan).

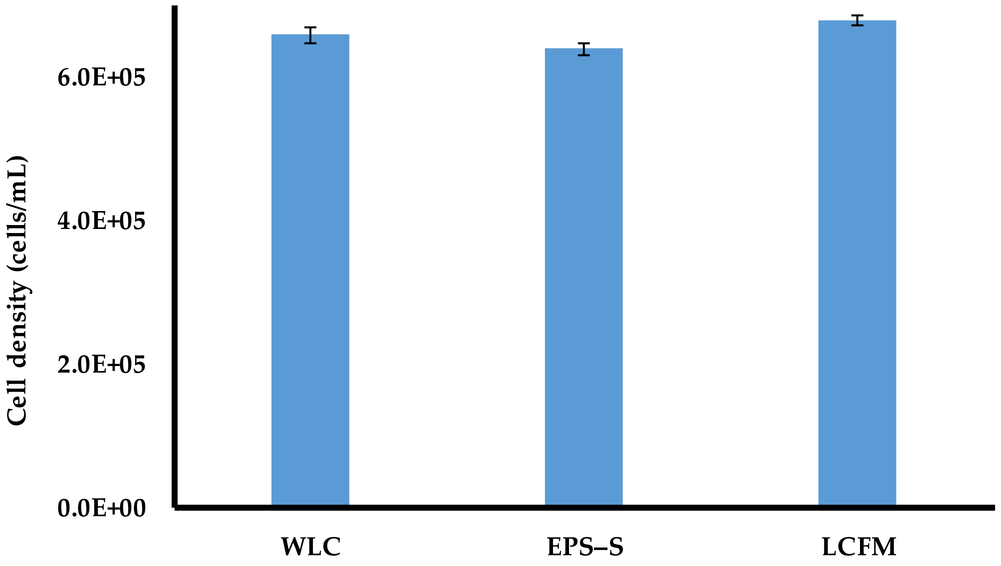

Figure S1. Cell density of *C. reinhardtii* cultures used in the three synthesis routes.

2. Cell culture chlorophyll *a* composition

Chlorophyll *a* was extracted from the cells by diluting 1 mL of cell culture with 9 mL of 99.5% acetone, which was then vortexed for 1 min using a Fisher Scientific Vortex Mixer (Fisher Scientific, Hampton, NH, USA), sonicated for 5 min using a Cole-Parmer Ultrasonic Cleaner (Cole-Parmer, Vernon Hills, IL, USA), incubated at 34–37 °C in water for 5 min and centrifuged at 2500× g for 5 min using an Ample Scientific F-33D Centrifuge (Ample Scientific LLC, Norcross, GA, USA). The supernatant was taken in 1.00 cm path length quartz cuvettes and scanned from 500 nm to 800 nm by a Cary-Varian 100 Bio UV-Visible Spectrophotometer (Agilent Technologies, Santa Clara, CA, USA). Finally, chlorophyll *a* concentration was calculated from the absorbance of the supernatant at 663 nm [1,2].

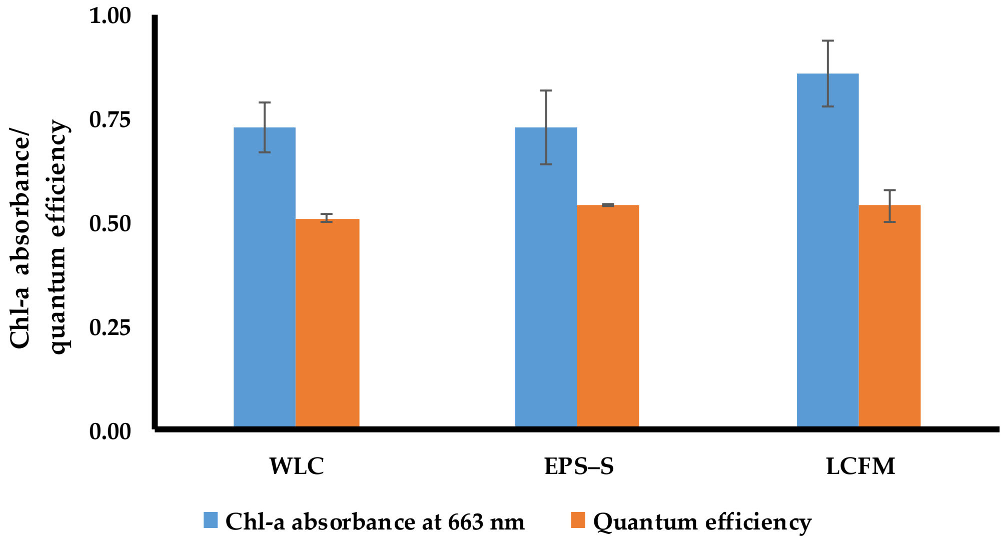

Figure S2. *Chl*-a absorbance at 663 nm and quantum efficiency of *C. reinhardtii* cultures used in the three synthesis routes.

3. Fluorescence signals and quantum efficiencies

Table S1a. Fluorescence signals (10× diluted) and quantum efficiencies (Q.E.) for WLC before and 1 h after the addition of $AgNO_3$.

Signal/ Q.E.	Initial values at 0 h	Values 1 h after the addition of $AgNO_3$			
		**0.000 mM**	**0.125 mM**	**0.625 mM**	**1.250 mM**
F_0	8290 ± 826	1291 ± 20	1161 ± 102	1179 ± 34	1148 ± 169
F_m	16364 ± 1556	2047 ± 16	1202 ± 88	1180 ± 39	1171 ± 185
F_v/F_m	0.49 ± 0.01	0.37 ± 0.01	0.03 ± 0.03	0.00 ± 0.00	0.02 ± 0.01

Table S1b. Fluorescence signals (10× diluted) and quantum efficiencies (Q.E.) for LCFM before and 1 h after the addition of $AgNO_3$.

Signal/ Q.E.	Initial values at 0 h	Values 1 h after the addition of $AgNO_3$			
		**0.000 mM**	**0.125 mM**	**0.625 mM**	**1.250 mM**
F_0	8772 ± 4331	11281 ± 230	8843 ± 821	6549 ± 749	6124 ± 612
F_m	17993 ± 7789	20872 ± 873	9027 ± 781	6604 ± 710	6134 ± 595
F_v/F_m	0.49 ± 0.05	0.46 ± 0.01	0.02 ± 0.01	0.01 ± 0.01	0.00 ± 0.00

Table S1c. Background noise from BBM control experiments 1 h after the addition of $AgNO_3$.

Signal	0 mM	0.125 mM	0.625 mM	1.250 mM
F_0	238	353	330	332
F_m	281	421	415	418

References

1. Whitney, D.E.; Darley, W.M. A method for the determination of chlorophyll *a* in samples containing degradation products1. *Limnol. Oceanogr.* **1979**, *24*, 183–186.
2. Shoaf, W.T.; Lium, B.W. Improved extraction of chlorophyll *a* and *b* from algae using dimethyl sulfoxide. *Limnol. Oceanogr.* **1976**, *21*, 926–928.
